# To Approach or to Avoid? Motivation Differentially Mediates the Effect of Hardiness on Depressive Symptoms in Chinese Military Personnel

**DOI:** 10.1155/2019/7589275

**Published:** 2019-05-27

**Authors:** Xiaoxia Wang, Janet Yuen-Ha Wong, Linkun Zhai, Ruicheng Wu, Tianhao Huang, Renqiang He, Yang Xiao, Yang Yu, Xiangji Kong, Xiaoyan Zhou, Hui Yang

**Affiliations:** ^1^Department of Basic Psychology, College of Psychology, Army Medical University, Chongqing 400038, China; ^2^School of Nursing, LKS Faculty of Medicine, The University of Hong Kong, Hong Kong; ^3^Cadet Brigade, Army Medical University, Chongqing 400038, China; ^4^Department of Clinical Psychology, The Mental Health Center of Chongqing, Chongqing 500000, China

## Abstract

**Objective:**

To investigate the mediation effect of approach/avoidance motivation between hardiness and depressive symptoms.

**Methods:**

Cross-sectional design was utilized. Two independent samples of military servicemen (G1: military personnel in the Armed Forces; G2: Chinese army military cadets) (n1 = 98, n2 =140) were sampled and investigated. The assessment tools of hardiness scale (DRS), behavioral activation and inhibition scales (BAS/BIS), and Center for Epidemiological Survey-Depression Scale (CES-D)/Beck Depression Inventory (BDI) were used. General linear model was conducted to examine the predictive role of hardiness (DRS) and motivation (BAS/BIS) on depressive symptoms (CES-D or BDI). The mediating role of BAS/BIS between hardiness and depressive symptoms was examined.

**Results:**

(1) Across army soldiers and military medical university cadets, hardiness (*β*=-0.394,* P*<0.001) and behavioral inhibition (*β*=0.297,* P*<0.001) significantly predicted depressive symptoms. (2) For soldiers only, behavioral inhibition mediated the significant association between hardiness and depressive symptoms (*β*=-0.043, SE=0.027, 95%CI=-0.130~-0.008). (3) For cadets only, behavioral activation-Drive significantly predicted depressive symptoms (*β*=-0.237,* P*=0.012), and hardiness operates through behavioral activation-Drive to influence depressive symptoms (*β*=-0.057, SE=0.036, 95%CI=-0.151~-0.078).

**Conclusion:**

Individuals who are low in hardiness and behavioral activation-Drive and who are high in behavioral inhibition showed more severe depressive symptoms. The relationship between hardiness and depressive symptoms was mediated by behavioral activation-Drive in cadets and behavioral inhibition in soldiers. The proposed model offers a useful approach for the development of hardiness training programs to alter approach/avoidance motivation in the military context. Future training program of hardiness could lay more emphasis on promotion of perseverance in pursuing goals in hardy individuals, which may in turn improve active coping.

## 1. Introduction

Conceptually, hardiness is deeply rooted in existentialism which emphasizes hardiness as commitment to challenges and motivation to cope with stressful circumstances to achieve meaning of life [1]. In the past few decades, an extensive body of researches on hardiness has been conducted in areas of clinical and military psychology [2]. Conceptually, hardiness is an individual disposition or style that remains relatively stable across cultures and could be shaped under training conditions. The construct of hardiness was first proposed by Kobasa [3] and then defined by Maddi as the constellation of three intercorrelated dimensions (3Cs):* Commitment* (the positive attitude, belief, and behavioral tendency exhibited by individuals who could engage life and work with commitment rather than retreating into isolation),* Challenge* (to see changes in life as challenges to grow and adjust effectively), and* Control* (to believe that they could exert control over the outcome) [4]. Across a range of occupational contexts and stressful conditions, converging evidence has indicated the buffering effect of hardiness against stress-related illness [3]. Greater level of psychological hardiness predicted adaptive immune and neuroendocrine responses to stress [5] and high level of happiness [6].

Military personnel are faced with stressful work situations including frequent deployments, family separation, life-threating missions, and long work hours. Hardiness has received attention in military populations for its protective role against stress and maintains a healthy and stable state. For example, in military context which involved high levels of deployment-related stress, hardiness is found to be related to less depression and posttraumatic stress disorder (PTSD) [7]. An important reason why hardy people are more effective in stressful situations is their active coping strategies [8]. Earlier studies showed that adaptive coping (e.g., problem-focused, support-seeking) and maladaptive coping (e.g., avoidant coping) mediated the hardiness-illness relationship [9]. More use of positive coping strategies (e.g., active coping and planning) and less use of negative coping strategies (e.g., behavioral disengagement) were identified in those active service members and veterans with greater hardiness [10, 11]. Specifically,* Commitment* enhanced mental health by the use of emotion-focused coping strategies.* Control* improved mental health by the use of problem-focused and support-seeking strategies [12]. Persons low in hardiness are more likely to use avoidant coping such as substance/alcohol abuse [13, 14] on the one hand and benefit from social cohesion and report less mental health problems on the other hand [15].

However, further evidence suggested that coping mediates the relationships between dispositional motivation and psychopathological symptoms [16]. Reinforcement sensitivity theory (RST) defined two motivation systems: (1) behavioral activation system (BAS), which guides approach motivation towards reward, and (2) behavioral inhibition system (BIS), which directs avoidance motivation away from punishment [17]. Furthermore, dispositional motivation is a distal predictor of behavior, while coping strategies may be seen as the proximal predictor of behavior [18]. High BIS sensitivity predisposed the individuals to recruit more attentional resources for detection of potential failure to obtain reward [19] and to adopt more avoidant coping strategies [18]. In contrast, BAS sensitivity played a protective role in working against maladaptive avoidant coping (e.g., gambling and alcohol use) [20]. Depression is characteristic of reduced BAS [21–23] and increased BIS [23, 24], or the combination of both [25]. Presumably, a coping style based on weak motivation to seek reward (lower BAS) and strong motivation to avoid punishment (higher BIS) may lead to increased feelings of depression. Mediational study could provide more insights about the relationship between hardiness and mental health outcome. Therefore, we proposed that approach/avoidance motivation may act as mediators between hardiness and depressive symptoms, which has not been tested directly. The aim of the current study is to (1) confirm the predicting effects of hardiness and BAS/BIS on depressive symptoms and (2) infer the mediating role of BAS/BIS between hardiness and depression symptoms.

## 2. Methods

### 2.1. Participants

To replicate the results within the relatively small sample, we conducted the investigation on another sample with similar age range. Therefore, two types of military personnel were investigated (G1: military personnel in the Armed Forces; G2: military medical university cadets).

*G1*. A group of military personnel in the Armed Forces were under survey (n=101; all male). The questionnaires with missing values (n=3) were excluded. Ninety-eight valid questionnaires were retained.

*G2*. A group of military medical university cadets were under survey (n=142; male: 137, female: 5). Those questionnaires with missing values (n=2) were excluded, with the remaining 140 valid questionnaires.

### 2.2. Measurements

The self-reported questionnaire included: (1) items on demographic and background variables, such as age, education, gender, and marital status, and (2) three measurement tools included DRS, BBS, CES-D (sample 1), or BDI (sample 2).

*(1) Dispositional Resilience Scale (DRS)*. DRS was originally developed by Paul Bartone [26] and was translated into Chinese version with satisfactory psychometric properties [27]. The DRS included 15 items and comprised of three dimensions (3Cs): Challenge, Commitment, and Control. The DRS was rated on a 4-point Likert scale ranging from 1 (not at all true) to 4 (completely true). The DRS has acceptable internal consistency (group 1: Cronbach alpha=0.607; group 2: Cronbach alpha=0.662). The 3-week test-retest reliability coefficient was 0.78 [26].

*(2) Behavioral Inhibition System and Behavioral Activation System Scale (BIS/BAS Scale, BBS)*. BBS was a reliable and valid instrument based on Gray's theory of reinforcement sensitivity [28]. The BBS was translated into Chinese and revised by Yanzhang Li [29]. The translated scale has 18 items and consists of 4 subscales: Behavioral Inhibition System (BIS) subscale (to measure avoidance motivation), Reward Responsiveness (RR), and Drive and the Fun Seeking (FS) subscale (to measure approach motivation). The RR, Drive, and FS subscales are comprised of behavioral activation system (BAS). The RR subscale measures the responsivity to current or anticipated positive stimuli. The Drive subscale measures the persistent pursuit of goals. The FS subscale measures the on-the-moment desire to obtain rewards or approach positive stimuli [28]. The scale uses a 4-point Likert scale ranging from “completely agree” to “completely disagree”. The 2-month test-retest reliability was 0.59~0.69 [29]. The DRS has good internal consistency (group 1: Cronbach alpha=0.843; group 2: Cronbach alpha=0.833).

*(3) The Center for Epidemiological Survey-Depression Scale (CES-D)*. CES-D was developed by the National Institute of Mental Health [30]. The CES-D aims to (1) screen for individuals with mild to severe depressive symptoms; (2) assess the severity of depressive symptoms in the past one week. The CES-D is comprised of 20 items which measure nine groups of depressive symptoms (*Sadness*,* Anhedonia*,* Loss of appetite*,* Sleep problems*,* Difficulty in thinking/concentration*,* Feelings of worthlessness*,* Fatigue*,* Agitation,* and* Suicidal ideation*) as defined by the DSM (Diagnostic and Statistical Manual) of APA (American Psychiatric Association). The CES-D is scored with a 4-point Likert scale ranging from 0 (“Not at all or less than one day”) to 3 (“5-7 days”). The range of possible scores of CES-D is between 0 (for those who respond 0 to all 20 questions) and 60 (for those who respond 3 to all 20 questions). People who have a total score of ≥16 but do not meet clinical criteria for major depressive episode are deemed as having possible/probable major depressive episode [30]. The CES-D has good internal consistency in the army soldiers (group 1: Cronbach alpha=0.763).

*(4) Beck Depression Inventory-II (BDI-II) Developed by Beck in 1967*. Similar to CES-D, it can be used to screen depression as well as for the assessment of the severity of depression in patients. It measures three components of depressive symptoms: (1) negative attitude or negative emotions such as pessimism and helplessness; (2) physical symptoms such as fatigue and sleep problems; (3) difficulties in operation with the feeling that work is more difficult than before. The BDI is scored with a four-point Likert scale ranging from 0 (never/rare) to 3 (very often), with total scores indicating the severity of depressive symptoms (0~13: no depression, 14~19: mild depression, 20~28: moderate depression, and 29~63: severe depression). The BDI-II has good internal consistency in the military medical university cadets (group 2: Cronbach alpha=0.913).

### 2.3. Procedure

All procedures were approved by Ethics Committee of Army Medical University (Chongqing, China). A group of military personnel in the Armed Forces (sample 1) and military medical university cadets (sample 2) were administered with paper and pencil questionnaires which assessed hardiness, approach/avoidance motivation and depressive symptoms. Before the test, seven undergraduate investigators were trained to become familiar with the test procedures, instructions and measurement tools. Verbal informed consent of all participants (sample 1) was obtained during July to August in 2015. Written informed consent of all participants (sample 2) was obtained during October of 2016 to May of 2017. Different informed consent forms and depression scales were utilized because two studies were conducted independently during 2015~2017. However, the documentation of the consent process including the names of all participants, information provided, and date consent obtained was kept in the study record. Participants completed the questionnaires in the classroom (sample 1) and laboratories (sample 2) with the same procedures and instructions, under the guidance of one investigator for each participant. This procedure may preclude the possibilities that the measures could be affected by the test condition. Furthermore, the use of two independent samples may allow us to draw robust conclusions about the effects found.

### 2.4. Statistical Analysis

Statistical Package for the Social Sciences version 22.0 (SPSS Inc., Chicago, IL) was used as the statistical software. General linear regression analyses were conducted with hardiness as independent variable, BAS, and BIS as mediating variables and depressive symptom (CES-D or BDI scores) as dependent variable. The predictive effect of independent variables (DRS, BAS, and BIS) on depressive symptoms (CES-D or BDI) was tested. PROCESS macro for SPSS [31] (model 4) were used, with hardiness as independent variable, BAS and BIS as mediating variables, and depressive symptoms (CES-D or BDI scores) as dependent variables. The bootstrap sampling method was adopted (resample size=1000) with 95% confidence interval to compute the indirect effect of BAS/BIS. The default setup of bootstrap sampling in SPSS macro PROCESS is 1000, which is sufficient for preliminary analyses. The results of mediation analysis were robust when 5000 sampling was utilized, which is the recommended number of sampling for final reporting (Preacher et al., 2008). These variables (hardiness, BAS/BIS, CES-D/BDI) were z-transformed and entered simultaneously into the regression analyses.

Then in order to verify the repeatability of the above results, the two data sets were combined. For convenience of direct comparison, the depressive symptom of the combined data set included standard score of the CES-D or BDI. The scores of other scales (including hardiness, BAS and BIS) are standardized. And then the above analyses were repeated on the assumption that the tested variables conform to the normal distribution.

## 3. Results

Descriptive statistics were listed below for army soldiers (sample 1) and military medical university cadets (sample 2). Between-group comparison of hardiness and motivation revealed that cadets had greater levels of hardiness and behavioral activation/inhibition than soldiers (Table 1). Specifically, greater commitment was shown in cadets than soldiers (t=-0.28,* P*=0.006), while challenge and control levels were comparable between groups (t=-0.83,* P*=0.41; t=1.08,* P*=0.28).

**Figure 1:**
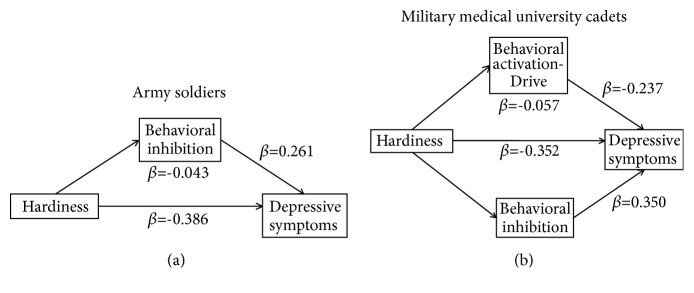
The mediating role of motivation between hardiness and depressive symptoms in military personnel.

The inferential statistical analyses (i.e., general linear regression and mediating analyses) were performed on the combined sample and then were repeated for sample 1 and 2, respectively. We explored the mediating role of subfactors of behavioral activation (Reward Responsiveness, Drive, and Fun Seeking), which were entered as mediators between hardiness and depressive symptoms. (1) For soldiers, behavioral inhibition was positively related to depressive symptoms (*β*=0.261,* P*=0.029), while hardiness negatively predict the depressive symptoms (*β*=-0.386,* P*<0.001). Behavioral inhibition (*β*=-0.043, SE=0.027, 95%CI=-0.130~-0.008) mediated the significant association between hardiness and depressive symptoms. (2) For cadets, behavioral inhibition was positively related to depressive symptoms (*β*=0.350,* P<*0.001), while hardiness and behavioral activation-Drive negatively predict the depressive symptoms (*β*=-0.352,* P*<0.001; *β*=-0.237,* P=*0.012). Behavioral activation-Drive (*β*=-0.057, SE=0.036, 95%CI=-0.151~-0.078) mediated the significant association between hardiness and depressive symptoms. (3) Across both groups, hardiness negatively predicted depression symptoms (*β*=-0.394,* P*<0.001), and behavioral inhibition positively predicted depressive symptoms (*β*=0.297,* P*<0.001) (Figure 1).

## 4. Discussions

The military occupation involves high risk, danger, and heavy workload. Hardiness prepares the personnel to cope actively and be resilient to military stress. The current study revealed that cadets had greater levels of hardiness and behavioral activation/inhibition than soldiers. Specifically, commitment was higher in cadets than soldiers. Furthermore, this study demonstrated that, (1) across both army soldiers and military medical university cadets, hardiness and behavioral inhibition significantly predicted depressive symptoms; (2) for soldiers only, behavioral inhibition mediated the significant association between hardiness and depressive symptoms; (3) for cadets only, behavioral activation-Drive significantly predicted depressive symptoms, and hardiness operates through behavioral activation-Drive to influence depressive symptoms.

### 4.1. Group Comparison of Hardiness and Behavioral Activation/Inhibition

Hardiness is a generalized style of functioning that includes cognitive, emotional, and behavioral features. Therefore, high hardy individuals tend to evaluate stress as challenge rather than threat. This adaptive cognitive style of hardy individuals manifested as cognitive flexibility during conscious and automatic emotion-regulatory processes [32]. The current study observed greater levels of hardiness in cadets which may enable these individuals to be more resilient to potentially threatening experiences and reduce the risk of or maintenance of depressive symptoms. However, the depressive symptoms of cadets were comparable to army soldiers which could be explained with the differentiated mediating role of motivation (e.g., behavioral activation/inhibition) of both groups. For example, for those who with greater hardiness, commitment to specific values and goals may enable the individuals to be actively engaged in life/work. The behavioral activation may lead to increasing rewarding experience which in turn helps alleviate the depressive symptoms [33]. Additionally, the sense of control in hardy individuals may help them realize the contingency between effort and reward, which makes these individuals tend to increase their efforts in the short term and increase the compensatory approach motivation to overcome the frustration associated with the sense of loss of control in the long term [34]. However, for those who with less hardiness, avoidance motivation as mediator may act as adaptive coping to alleviate the detrimental effect of aversive environment stimuli and decrease the risk of depressive symptoms.

### 4.2. Mediation Model between Hardiness and Depressive Symptoms

*First*, this study confirmed the mediating rather than predictive role of approach motivation on depressive symptoms in military personnel. The approach motivation has an effect on depressive symptoms with group specificity. For the sample of military medical university cadets rather than army soldiers, behavioral activation-Drive mediated the relationship between hardiness and depressive symptoms. Sensitivity to rewards (BAS) (esp. Fun Seeking and Reward Responsiveness) plays an important role in cognitive processing (e.g., updating and working memory maintenance) of potential reward stimuli [19]. Furthermore, individuals with greater behavioral activation-Drive exhibit stronger motivation to pursue goals, regardless of whether these goals are inherently pleasurable [28]. This may enhance the opportunities to response-contingent positive reinforcement and lead to positive affect and wellbeing [35]. This result may help explain why hardiness could be enhanced through systematic training by choosing controllable goals and effective skills to deal with challenges [36, 37]. Therefore, the role of behavioral activation, especially the persistent pursuit of goals, should be emphasized during hardiness training of army soldiers. However, stronger trait avoidance motivation is also associated with increased risk of onset and chronicity of depressive disorders [24], which suggested the significance of inflexible coping strategies in less hardy individuals.

*Second*, this study found that the avoidance motivation mediated the relationship between hardiness and depressive symptoms in army soldiers, while positively predicted depressive symptoms in military medical cadets. Moderate level of sensitivity to punishment (BIS) may predispose the depressed individuals to negative attention bias towards negative stimuli and greater approach behavior towards disliked activities [19, 38]. In contrast, individuals with greater avoidance motivation may be more directly related to experience negative affect [35], negative cognitive/physiological reactivity [39], and depressive symptoms [40]. Meanwhile, BAS sensitivity (Drive) may work against the protective role of hardiness towards depressive symptoms. Therefore, the reduction of avoidance motivation is also an important target for evidence-based Behavioral Activation Treatment of Depression (BATD), which helps the depressed military personnel to set goals of personal value and surmount obstacles and finally to experience personal rewards after goal-attainment [41, 42]. Accordingly, training programs to increase hardiness and decrease avoidance motivation in high-stress occupations such as the military are surely needed.

*Limitations*. The results of the present study suggest that hardiness is an important variable contributing to depressive symptoms of soldiers and military medical cadets, with avoidance motivation as potential mediating factor. One major limitation is the cross-sectional nature of this study. Participants answered questions regarding hardiness, motivation, and depressive symptoms at the same time, which may inflate the correlations between these variables. Therefore, longitudinal study is needed to verify the main findings of this study. Although these results are suggestive regarding the underlying mediating processes through which hardiness affects depressive symptoms, the robustness of the observed relationships between hardiness, motivation, and depressive symptoms was evidenced in at least two types of regression (linear; logistic). Nonetheless, replication of these findings in separate demographically similar samples (such as Army Reserve Medical Unit and Special Forces) and higher stress situations is needed for generalization of the results. Another limitation of the study is the relatively modest sample size, although it met the standard of mediation test proposed by Fristz and MacKinnon [43]. However, future study with larger sample size is combined with advanced statistical models including stress variables to examine the stress-resilience assumption.

## Figures and Tables

**Table 1:** The hardiness personality, avoidance motivation, and depressive symptoms of the military community.

	Soldiers (M±S.D.)	Cadets (M±S.D.)	t (*P*)
Age	15~30^a^	21.30 ± 1.89	--
Gender (male/female)	98/0	135/5	--
Education (middle school/high school/junior college/university)	26/64/7/1	0/0/0/140	--
DRS	41.58±5.81	43.20±4.05	-2.38*∗*
BAS-drive	11.33±2.27	12.40±1.84	-3.85*∗∗∗*
BAS-reward responsiveness	12.35±2.04	13.32±1.62	-4.07*∗∗∗*
BAS-fun seeking	13.72±2.69	15.03±1.98	-4.08*∗∗∗*
BIS	13.50±3.25	18.74±2.95	-12.91*∗∗∗*
Z_CES-D/Z_BDI	-0.0007±1.01	0.004±1.00	-0.04

Note: *∗P*<0.05, *∗∗P*<0.01, and *∗∗∗P*<0.001. ^a^ The age range (1=*15~20 years*; 2=*21~25 years*; 3=*26~30 years*; 4=*31~35 years*; 5=*36 years and above*) was collected. DRS=Dispositional Resilience Scale; BAS=Behavioral Activation Scale; BIS=Behavioral Inhibition Scale; Z_CES-D=Z value of CES-D (Center for Epidemiological Survey Depression Scale) total score; Z_BDI=Z value of BDI (Beck Depression Inventory) total score.

## Data Availability

The data studied is available from the authors upon request.
